# Evaluation of femtosecond laser-assisted anterior capsulotomy in the presence of ophthalmic viscoelastic devices (OVDs)

**DOI:** 10.1038/s41598-020-78361-8

**Published:** 2020-12-09

**Authors:** Hassan Mansoor, Yu-Chi Liu, Yoke Rung Wong, Nyein C. Lwin, Xin Y. Seah, Jodhbir S. Mehta

**Affiliations:** 1 Tissue Engineering and Stem Cell Group, Singapore Eye Research Institute, Singapore, Singapore; 2 Singapore National Eye Centre, 11 Third Hospital Avenue, Singapore, 168751 Singapore; 3 Biomechanics Laboratory, Singapore General Hospital, Singapore, Singapore; 4 Al-Shifa Trust Eye Hospital, Rawalpindi, Pakistan; 5 Ophthalmology Academic Clinical Program, Duke-NUS Graduate Medical School, Singapore, Singapore; 6 School of Material Science and Engineering, Nanyang Technological University, Singapore, Singapore

**Keywords:** Translational research, Surgery

## Abstract

The introduction of femtosecond laser-assisted cataract surgery is an alternative approach to conventional cataract surgery. Our study aimed to determine the effectiveness of femtosecond laser-assisted capsulotomy in the presence of different ophthalmic viscoelastic devices (OVDs) in the anterior chamber. Fresh porcine eyes (n = 96) underwent LDV Z8-assisted anterior capsulotomy, either in the presence of an OVD (Viscoat, Provisc, Healon, Healon GV or HPMC) or without, using 90% and 150% energies respectively. Following that, the capsule circularity, tag’s arc-length, tag-length, tag-area and rupture strength (mN) of the residual capsular bag were evaluated. We found that increasing energy from 90 to 150% across the OVD sub-groups improved the studied capsulotomy parameters. Amongst the 90% energy sub-groups, the circularity and tag-parameters were worse with Viscoat and Healon GV, which have higher refractive index and viscosity compared to the aqueous humour. Using 150% energy, Healon GV showed a significantly worse total arc-length (p = 0.01), total tag-length (p = 0.03) and total tag-area (p = 0.05) compared to the control group. We concluded that; an OVD with a refractive index similar to aqueous humour and lower viscosity, such as Healon or Provisc, as well as a higher energy setting, are recommended, to enhance the efficacy of laser capsulotomy.

## Introduction

Femtosecond laser-assisted cataract surgery (FLACS) has been introduced over the last decade as an alternative to conventional cataract surgery^1^. The potential benefits of FLACS include automation of certain steps in the cataract surgery procedure, i.e. anterior capsulotomy, nuclear fragmentation and corneal incisions, in a precise and a highly reproducible manner^2,3^. Pre-segmentation of the cataractous lens, by a femtosecond laser (FSL), has been shown to reduce phacoemulsification time and energy^4–6^. Moreover, some studies have suggested that the anterior capsulotomy strength, produced by certain FSL, may exceed that obtained with a manual capsulorhexis^4,7^. These features maybe particularly useful in improving the safety profile of cataract surgery in patients with pseudoexfoliation syndrome, small eyes (axial length < 21 mm), Fuchs endothelial corneal dystrophy, floppy iris syndrome, subluxated lens, hard nucleus, and patients with poor red reflex^4^. However, the financial cost of FSL platforms, and lack of superior visual and refractive outcomes in comparison to traditional phacoemulsification has limited its widespread adoption^2,8^.

In cataract surgery, either in FLACS or conventional phacoemulsification, a small pupil makes the surgery more difficult, due to the reduced visibility of the anterior lens capsule, lack of red reflex and limited space for intraoperative manipulation^4,9^. It also increases the intraoperative risk of iris trauma, retained lens matter and posterior capsule rupture^9^. Poor pupil dilation may be found in patients with diabetes, pseudoexfoliation, senile atonic pupils, posterior synechiae, long-term pilocarpine usage and trauma^4,9^. A limitation of all current FSL platforms is the prerequisite for patients to have adequately dilated pupils^2^. The minimum pupillary diameter required to proceed with FLACS varies amongst different FSL platforms. It is 4 mm and 3.5 mm for Victus (Bausch & Lomb, Inc. USA) and LDV Z8 (Ziemer Ophthalmic Systems AG, Switzerland) respectively, while Catalys (Optimedica Corp, Santa Clara, California, USA) has no definite minimum requirement^2^. Some surgeons may use intracameral epinephrine solution, viscomydriasis and/or mechanical pupil-expanding devices (e.g. Malyugin ring, iris hooks, etc.), to enable adequate pupillary dilation for femtosecond anterior capsulotomy^9–12^. A 3-step sequential approach has been suggested, with the use of intracameral epinephrine (0.1% solution), viscomydriasis and subsequent mechanical pupil expansion, if needed^12^.

Ophthalmic viscoelastic devices (OVDs) have been shown to aid the insertion of mechanical pupil expanders and to be used in viscomydriasis^10,11^. The most commonly used OVDs in cataract surgery include Viscoat (Alcon, Inc. USA), Healon (Advanced Medical Optics, Inc. USA), Provisc (Alcon, Inc. USA), Healon GV (Johnson & Johnson Surgical Vision, Inc. USA) and HPMC (Rayner Intraocular Lenses Limited, UK), which differ in their physical and chemical properties^13–15^. The OVDs mentioned above have a refractive index (*n*) close to aqueous humour (*n* = 1.336), other than Viscoat (Alcon, Inc. USA), which has a higher refractive index (*n* = 1.342)^16^. Differences in refractive indices and viscosity amongst OVDs may alter the effectiveness of the FSL, during anterior capsulotomy and nuclear fragmentation^10–12^. Dick and Schultz used mechanical pupil expanders, with or without an OVD, to achieve adequate pupillary dilation in FLACS, using the Catalys (Optimedica Corp, Santa Clara, California, USA)^11^. Incomplete capsulotomies and/or capsular tags were more common when the anterior chamber (AC) was filled with an OVD during the laser treatment^11^. The authors hypothesized that the difference in viscosity between aqueous humour and an OVD could affect the cutting-depth range of the FSL beam and result in an incomplete anterior capsulotomy^11^. Hence, they suggested increasing the laser power or elevating the laser cut-depth to avoid this issue^11^. However, there are no clinical studies, supporting these recommendations.

Recently, an optical model of the anterior segment, based on the schematic Gullstrand eye model, was used to simulate conditions during FLACS^16^. Using this model, the refractive index (*n*) of aqueous humour and of the OVD, posterior radius of corneal curvature, and the anterior chamber depth were used in mathematical calculations to predict the error in cutting-depth of the FSL beam, when the AC was filled with an OVD^16^. The authors showed that the change in the refractive index (*n*) of the AC had a minimal effect on the depth of FSL-cut (− 8 μm to + 13 μm), and hence, was unlikely to result in an incomplete capsulotomy^16^. The error was considered negligible since the anterior capsule thickness (8–20 μm), and safety margins of different FSL platforms were found to be larger than the predicted error^16^. Additionally, the authors suggested that altering the laser power and/or laser-cut depth would not yield additional benefits^16^. However, the authors didn’t take into account the change in viscosity, caused by the AC refilling with an OVD, which may also influence the energy threshold requirement, for cavitation.

Hence, it is yet to be determined if the findings from the optical model of the anterior segment apply to a real-life clinical scenario^16^. It remains uncertain how the difference in refractive index (*n*), viscosity, cohesion and dispersion amongst different OVDs affect the energy requirements for successful femtosecond anterior capsulotomy. Therefore, this study aimed to evaluate the effectiveness of femtosecond laser-assisted capsulotomy, with the Ziemer LDV Z8, in the presence of different OVDs in the AC.

## Results

### Central corneal thickness (CCT) and anterior chamber depth (ACD)

A pooled analysis showed no significant difference in the CCT before and after the application of FSL across the sub-groups with 90% energy (0.81 ± 0.03 mm vs 0.82 ± 0.06 mm, p = 0.12) and 150% energy (0.81 ± 0.05 mm vs 0.83 ± 0.08 mm, p = 0.20).

Prior to OVD injection, there was no statistically significant difference amongst both the sub-groups (p = 0.25, p = 0.61) in ACD. The increase in the ACD post-OVD injection was also comparable between the sub-groups, (90% energy, p = 0.26), (150% energy, p = 0.60). Likewise, a comparison of ACD in both sub-groups, post-OVD injection, and post-FSL treatment; revealed no significant difference (90% energy p = 0.09 and p = 0.45 respectively; 150% energy p = 0.28 and p = 0.92 respectively; Table 1).

**Table 1:** Comparison of anterior chamber depth (ACD) in different sub-groups with 90% and 150% energy settings.

Group	Pre-OVD ACD	Post-OVD ACD	Increase in ACD after OVD injection	p value (pre-OVD ACD vs post-OVD ACD)	Post-FSL ACD	Difference in ACD before and after FSL treatment	p value (post-OVD ACD vs post-FSL ACD)
90% No OVD	2.54 ± 0.18	2.54 ± 0.18	0.00 ± 0.00		2.54 ± 0.18	0.00 ± 0.00	
90% Viscoat	2.45 ± 0.15	3.29 ± 0.29	0.84 ± 0.31	< 0.001	3.06 ± 0.32	0.23 ± 0.12	0.001
90% Provisc	2.59 ± 0.27	3.26 ± 0.18	0.67 ± 0.37	0.001	2.95 ± 0.22	0.31 ± 0.25	0.010
90% Healon	2.69 ± 0.21	3.54 ± 0.31	0.84 ± 0.23	< 0.001	3.20 ± 0.30	0.34 ± 0.24	0.008
90% Healon GV	2.49 ± 0.30	3.40 ± 0.33	0.91 ± 0.21	< 0.001	2.98 ± 0.38	0.41 ± 0.20	0.001
90% HPMC	2.48 ± 0.19	3.52 ± 0.17	1.04 ± 0.32	< 0.001	3.10 ± 0.25	0.42 ± 0.24	0.001
p value	0.25	0.09*	0.26*		0.45*	0.41*	
150% No OVD	2.58 ± 0.26	2.58 ± 0.26	0.00 ± 0.00		2.58 ± 0.26	0.00 ± 0.00	
150% Viscoat	2.53 ± 0.27	3.24 ± 0.26	0.71 ± 0.24	< 0.001	3.08 ± 0.30	0.18 ± 0.09	0.010
150% Provisc	2.62 ± 0.22	3.41 ± 0.24	0.79 ± 0.22	< 0.001	3.10 ± 0.12	0.32 ± 0.23	0.008
150% Healon	2.67 ± 0.21	3.36 ± 0.31	0.68 ± 0.31	< 0.001	3.08 ± 0.43	0.27 ± 0.18	0.004
150% Healon GV	2.74 ± 0.28	3.55 ± 0.33	0.81 ± 0.16	< 0.001	3.27 ± 0.42	0.28 ± 0.23	0.009
150% HPMC	2.56 ± 0.22	3.48 ± 0.28	0.91 ± 0.45	< 0.001	3.11 ± 0.37	0.39 ± 0.19	0.004
p value	0.61	0.28*	0.60*		0.92*	0.37*	

p values comparing the groups in the presence of different types of OVD.

### Efficacy of laser capsulotomy

All the eyes in the control groups had complete capsulotomies after FSL treatment. However, incomplete capsulotomies were seen with 90% and 150% energy settings, when AC was filled with an OVD during the laser treatment. A lower energy setting was twice more likely associated with an incomplete capsulotomy (40 ± 13.69% vs 20 ± 11.18%, p = 0.06). The percentage of incomplete capsulotomies in 90% and 150% energy sub-groups is shown in Table 2.

**Table 2:** Percentage of incomplete laser capsulotomies in different sub-groups with 90% and 150% energy settings.

Groups	90% Energy (*)	150% Energy (*)	p value
No OVD	0.0% (0/8)	0.0% (0/8)	–
Viscoat	25.0% (2/8)	12.5% (1/8)	0.52
Provisc	50.0% (4/8)	25.0% (2/8)	0.30
Healon	25.0% (2/8)	12.5% (1/8)	0.52
Healon GV	50.0% (4/8)	37.5% (3/8)	0.61
HPMC	50.0% (4/8)	12.5% (1/8)	0.11
Mean ± SD	40 ± 13.69%	20 ± 11.18%	0.06

*The numerator represents the incomplete laser-cuts, whereas the denominator denotes the total number of samples in each sub-group.

### Clinical grading of laser capsulotomy

**Figure 1:**
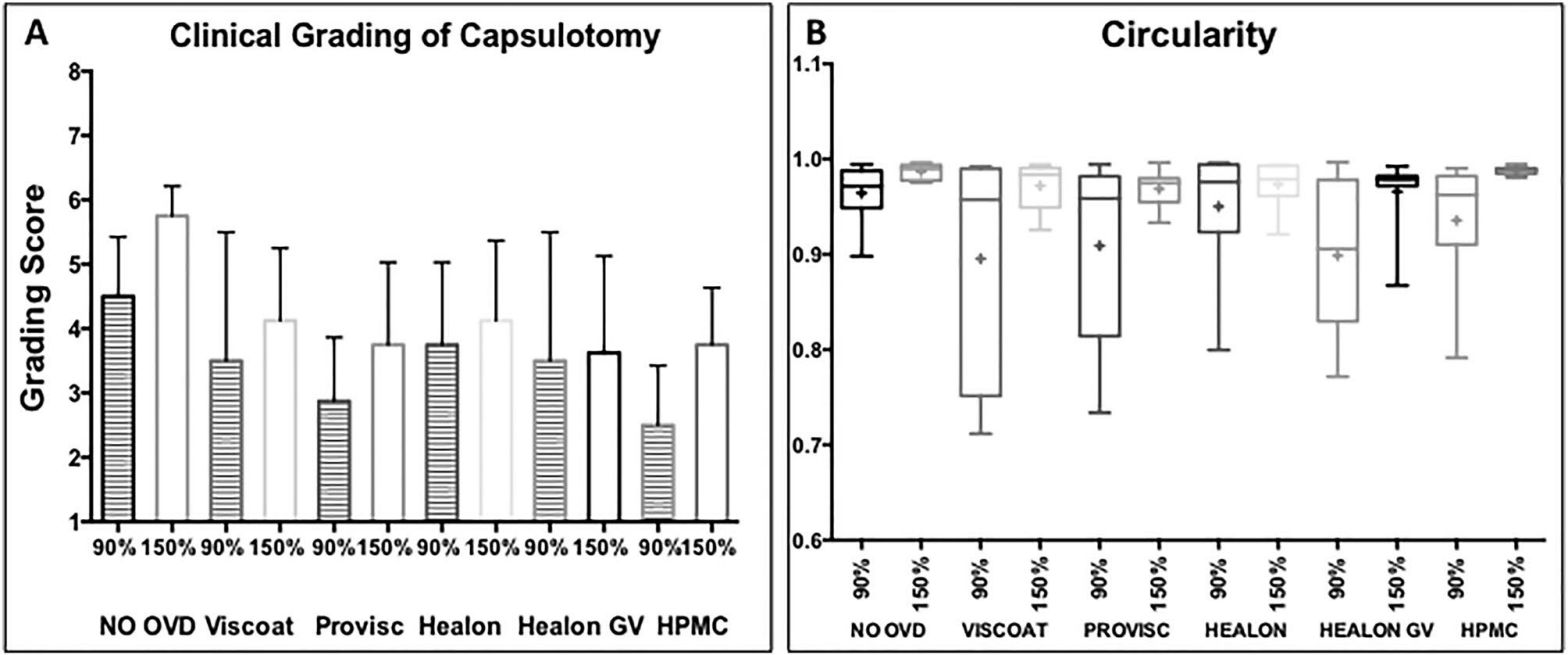
Comparison of the clinical grading of laser-capsulotomy and circularity in sub-groups with 90% and 150% energy settings. (**A**) The striped and plain bar charts represent the grading score, with 90% and 150% energy settings, respectively. (**B**) The box and whisker plots show variability in the plotted data for circularity. The ‘mean’ is represented by a ‘+’ in the box plots. The box extends from the 25th to 75th percentiles. (**A**,**B**) Increasing energy setting from 90 to 150% improved the grading score of laser-capsulotomy, and showed a better circularity, respectively, across the sub-groups.

The clinical grading of laser capsulotomy in all the sub-groups improved with a higher energy setting compared to a lower energy (p = 0.009, Fig. 1A). There was no significant difference in the clinical grading of capsulotomy amongst the 90% energy sub-groups (p = 0.09). While using 150% energy, the grading score achieved with Viscoat, Provisc, Healon, Healon GV and HPMC was significantly worse compared to the control group (p = 0.003, p = 0.0005, p = 0.004, p = 0.0005 and p = 0.0004 respectively).

### Capsule circularity

**Figure 2:**
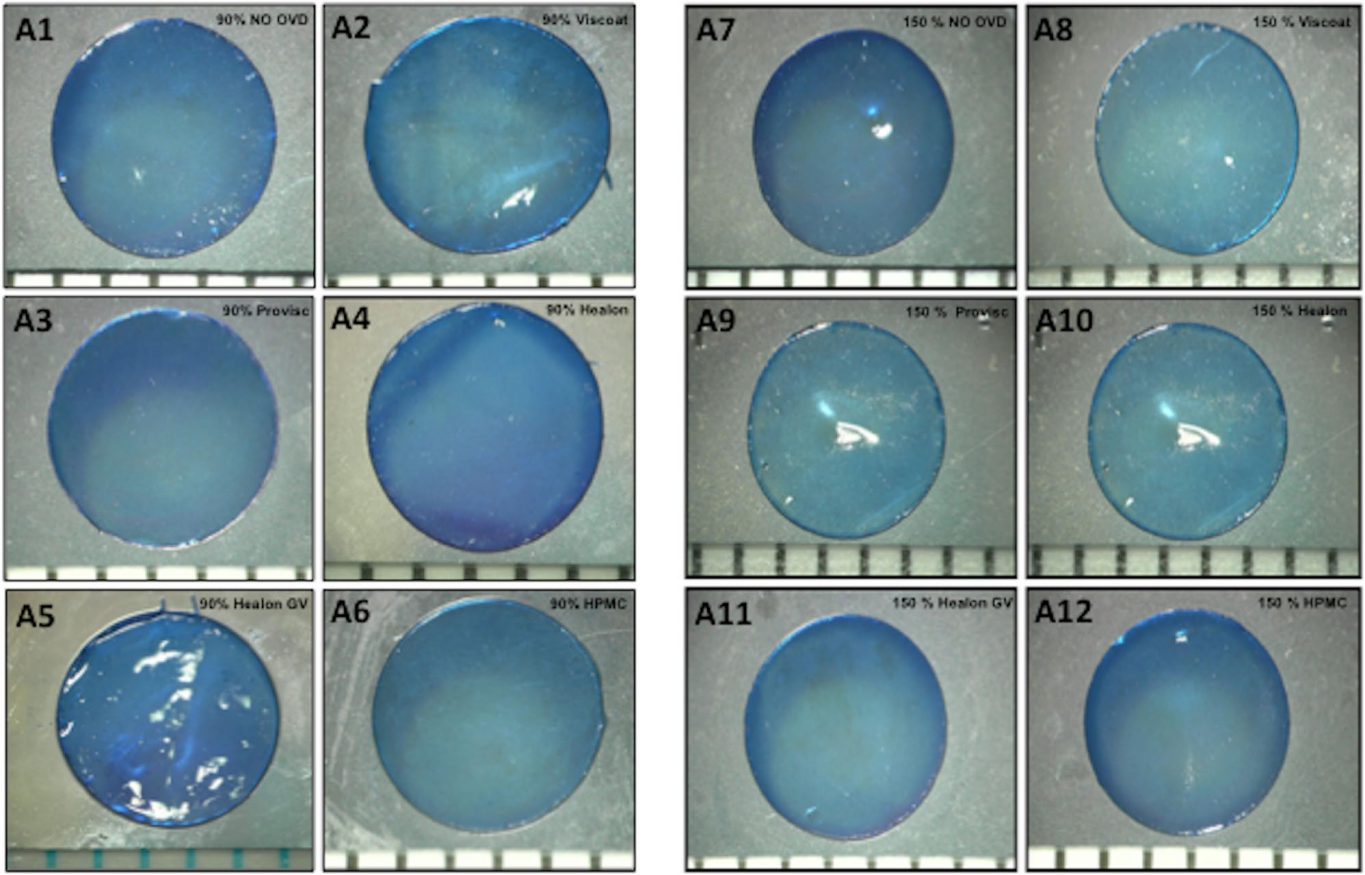
Explanted porcine lens-capsules. The figure shows explanted porcine capsules, which were stained with trypan blue (0.1%), and represent the mean circularity of different sub-groups, with (**A1**–**A6**) 90% energy and (**A7**–**A12**) 150% energy, respectively.

Representative capsule images of different sub-groups and comparison of the circularity achieved with 90% and 150% energy settings are shown in Fig. 2. Overall, increasing energy setting from 90 to 150% showed a significantly better circularity (0.92 ± 0.01 vs 0.97 ± 0.01; p = 0.0001, Fig. 1B). Using 90% energy, the capsule circularity was decreased in the presence of Viscoat (0.89 ± 0.12) and Healon GV (0.89 ± 0.15) compared to the control (0.96 ± 0.03) and other OVD sub-groups (Provisc; 0.91 ± 0.09, Healon; 0.95 ± 0.07, HPMC; 0.93 ± 0.06, p = 0.74) (Fig. 2). The circularity of Healon GV sub-group (0.96 ± 0.04) was worse in comparison to other sub-groups even at 150% energy setting (Control; 0.99 ± 0.01, Viscoat; 0.97 ± 0.02, Provisc; 0.97 ± 0.02, Healon; 0.97 ± 0.02, HPMC; 0.99 ± 0.01, p = 0.10) (Fig. 2).

### The number of tags

**Figure 3:**
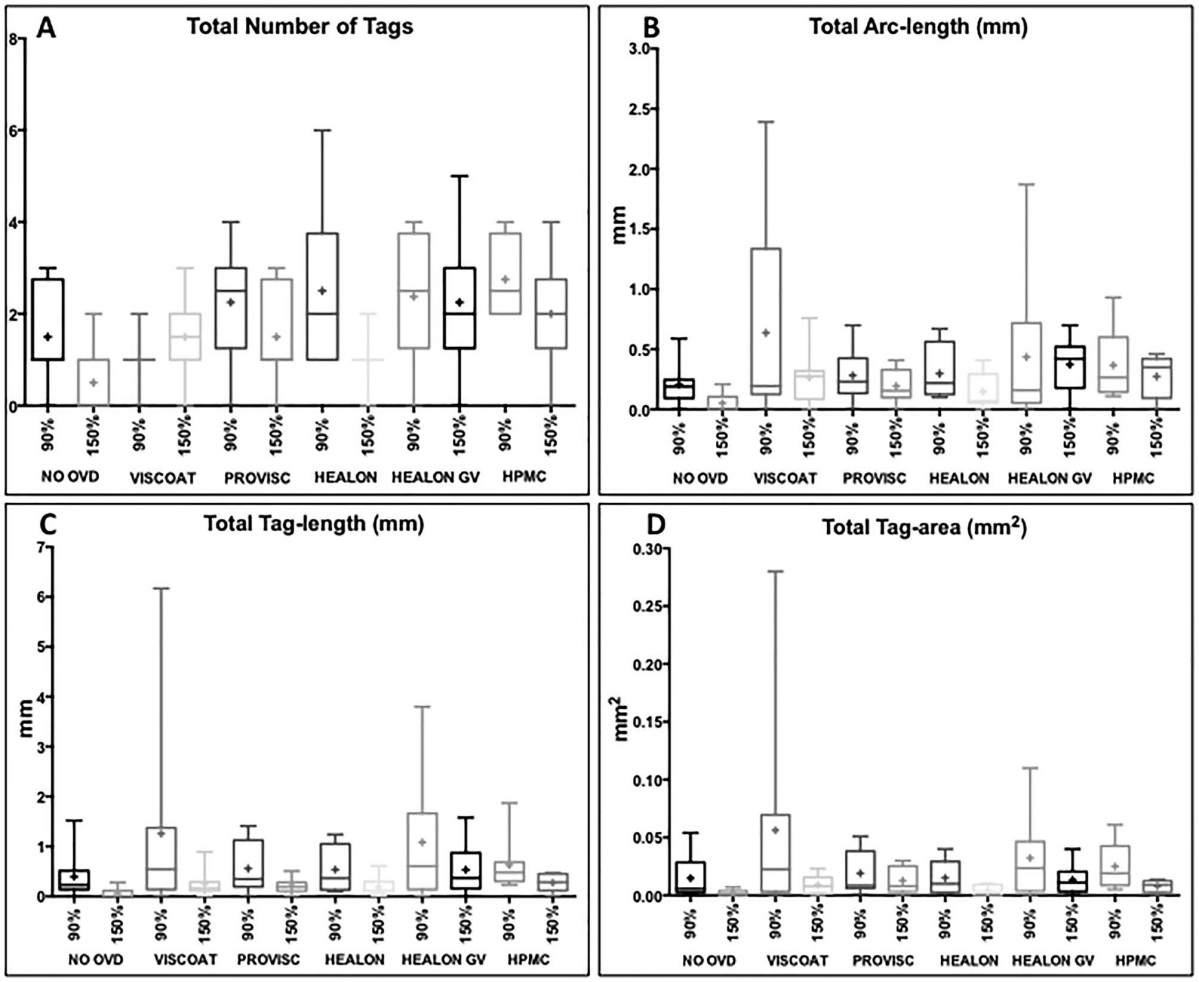
Comparison of total number of tags, total arc-length, total tag-length and total tag-area in sub-groups, with 90% and 150% energy settings. (**A**–**D**) The box and whisker plots showing variability in the plotted data for the total number of tags, total arc-length, total tag-length and total tag-area, respectively. A higher energy setting (150%) reduced the total number of tags, decreased the total arc-length and total tag-length, as well as reduced the total tag-area, respectively, across the sub-groups. The ‘mean’ is represented by a ‘+’ in the box plots. The box extends from the 25th to 75th percentiles.

A comparison of the total number of tags in different sub-groups, with 90% and 150% energy settings, is shown in Fig. 3A. It was noted that the total number of tags reduced in all the sub-groups after increasing the energy setting from 90 to 150% (p = 0.04). Amongst 90% energy sub-groups, HPMC sub-group (2.75 ± 0.89) displayed the highest number of tags (p = 0.03). With 150% energy, Healon GV sub-group (2.25 ± 1.48) showed the highest number of tags, with a significant difference compared to the control group (0.50 ± 0.75) (p = 0.02).

### Arc-length of tags

Increasing energy setting from 90 to 150% across the sub-groups recorded a borderline significant decrease in the total arc-length of tags (p = 0.06; Fig. 3B). There was no significant difference in the total arc-length amongst the 90% energy sub-groups (Control: 0.21 ± 0.17 mm, Viscoat: 0.64 ± 0.88 mm, Provisc: 0.28 ± 0.22 mm, Healon: 0.29 ± 0.24 mm, Healon GV: 0.44 ± 0.64 mm and HPMC: 0.37 ± 0.26 mm, p = 0.57). However, the total arc-length achieved with Healon GV (0.37 ± 0.22 mm) was significantly worse compared to the control group (0.05 ± 0.08 mm), with 150% energy (p = 0.01).

### Tag-length

The use of a higher energy setting compared to lower energy significantly reduced the total tag-length in all the sub-groups (p = 0.004) as shown in Fig. 3C. A comparison of total tag-length in 90% energy sub-groups revealed that the performance of Viscoat and Healon GV was the worst (p = 0.53) (Control: 0.39 ± 0.48 mm, Viscoat: 1.25 ± 2.04 mm, Provisc: 0.56 ± 0.52 mm, Healon: 0.54 ± 0.46 mm, Healon GV: 1.08 ± 1.28 mm and HPMC: 0.63 ± 0.53 mm). Amongst 150% energy sub-groups, Healon GV sub-group was significantly worse than the control group (p = 0.03; Control: 0.06 ± 0.10 mm, Viscoat: 0.25 ± 0.27 mm, Provisc: 0.21 ± 0.15 mm, Healon: 0.18 ± 0.20 mm, Healon GV: 0.53 ± 0.52 mm and HPMC: 0.27 ± 0.17 mm, p = 0.04). The longer tags in terms of the total tag-length were more frequently encountered with 90% energy setting (41.7% vs 8.3%; p < 0.001).

### Tag-area

**Figure 4:**
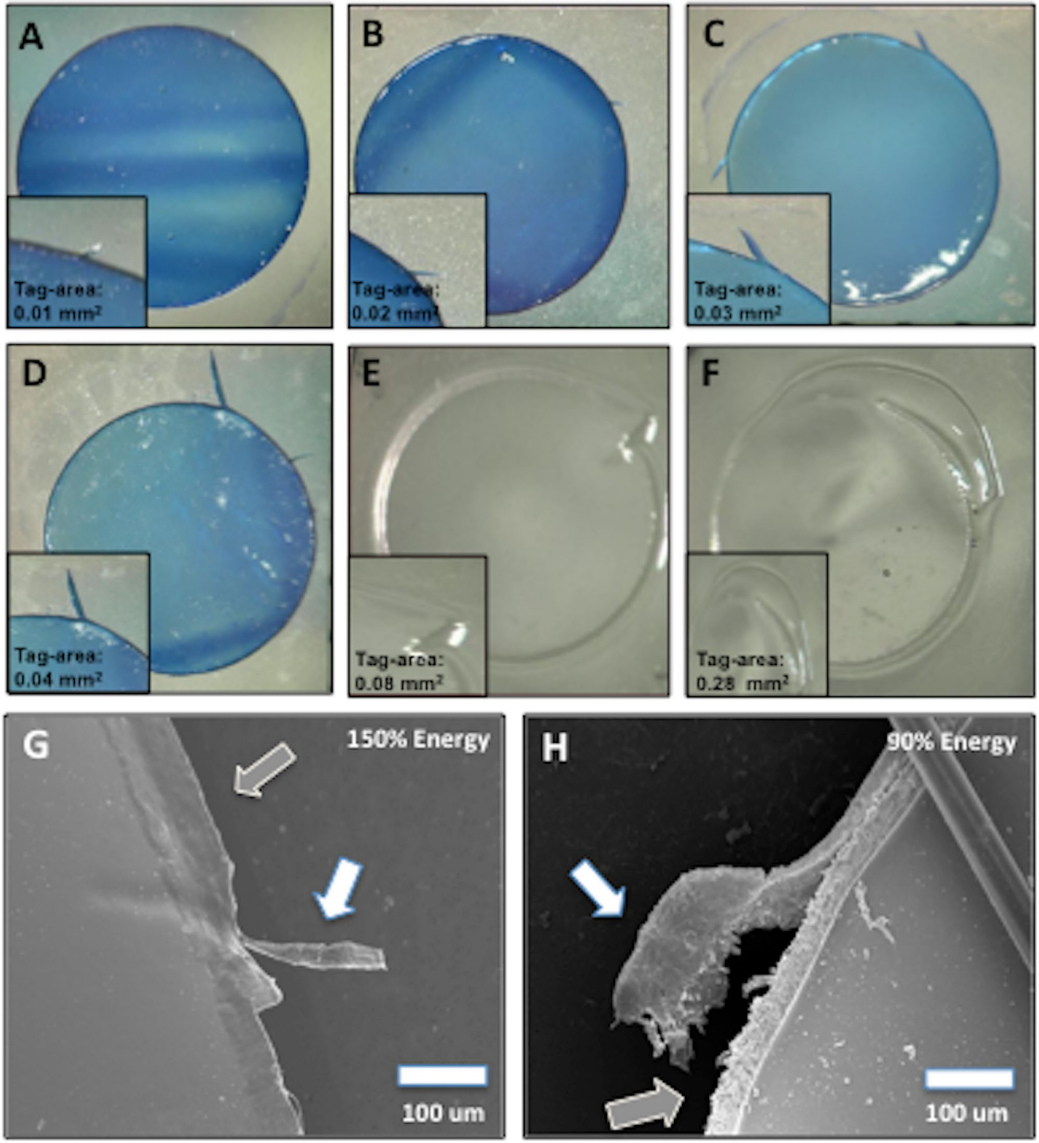
Lens capsules showing severity of a capsular-tag based on tag-area and SEM images of capsule edge morphology. (**A**,**B**) Complete laser-cut with macroadhesion. (**C**,**D**) Incomplete laser-cut with macroadhesions. (**E**,**F**) Non-continuous/irregular laser-cut showing worst tag-area. (**G**,**H**) High magnification (× 1000) SEM images of capsule-edge. The white arrow highlights the (**G**) small-tag and (**H**) big-tag respectively. The grey arrow shows the (**G**) relatively smooth and (**H**) rough capsule edge, seen with 150% and 90% energy settings, respectively.

Overall, the total tag-area reduced significantly with an increased (150%) energy setting (0.03 ± 0.04 mm^2^ vs 0.01 ± 0.01 mm^2^; p = 0.005, Fig. 3D). An analysis of 90% energy sub-groups revealed that the total tag-area attained with Viscoat (0.05 ± 0.09 mm^2^) and Healon GV (0.03 ± 0.04 mm^2^) was larger in comparison to other sub-groups (Control: 0.01 ± 0.02 mm^2^, Provisc: 0.02 ± 0.02 mm^2^, Healon: 0.01 ± 0.01 mm^2^, HPMC: 0.02 ± 0.02 mm^2^, p = 0.75). When using 150% energy, the total tag-area (mm^2^) observed with Healon GV (0.013 ± 0.01 mm^2^) was still larger compared to other sub-groups (Control: 0.001 ± 0.002 mm^2^, Viscoat: 0.01 ± 0.01 mm^2^, Provisc: 0.01 ± 0.01 mm^2^, Healon: 0.004 ± 0.004 mm^2^, HPMC: 0.007 ± 0.005 mm^2^, p = 0.05). Representative images of the capsular tags showing their severity based on tag-area are shown in Fig. 4A–F.

A lower energy setting (90%) in the presence of an OVD resulted in a significantly higher risk of creating a ‘large tag’, with respect to total tag-area (p = 0.007), along with an increased risk of a clinically significant tag (p = 0.001), during the laser treatment. The occurrence of a large tag, as well as a clinically significant tag, was independent of the type of an OVD used, with both 90% (p = 0.98 and p = 0.44 respectively) and 150% energies (p = 0.26 and p = 0.40 respectively).

### Correlation between tag parameters

There was a significant, moderate and inverse correlation between the circularity and total arc-length (r =  − 0.66, p < 0.001), circularity and total tag-length (r =  − 0.76, p < 0.001) as well as circularity and total tag-area (r =  − 0.71, p < 0.001). A significantly strong and positive correlation was seen between the total arc-length and total tag-area (r = 0.84, p < 0.001), total arc-length and total tag-length (r = 0.89, p < 0.001), and total tag-area and total tag-length (r = 0.94, p < 0.001).

### Capsulotomy strength and stretch ratio

There was an insignificant difference in the capsulotomy strength across the sub-groups after increasing the energy from 90 to 150% (187.46 ± 42.09 mN vs 181.31 ± 40.92 mN, p = 0.47). However, a significant difference was observed in the capsulotomy strength of 90% energy sub-groups (p = 0.002) with Viscoat displaying the worst capsulotomy strength (143.6 ± 69.77 mN). No significant difference was detected in the capsulotomy strength of 150% energy sub-groups (p = 0.72) (Table 3). The stretch ratio was not significantly different across the sub-groups while using an increased energy setting (p = 0.54). The stretch ratio was comparable in 90% energy sub-groups (p = 0.07) and 150% energy sub-groups (p = 0.42) as shown in Table 4.

**Table 3:** Comparison of capsulotomy strength (mN) in different sub-groups with 90% and 150% energy settings.

Groups	90% Energy	150% Energy	p value
No OVD	183.1 ± 22.38	177.1 ± 0.36	0.60
Viscoat	143.6 ± 69.77	181.8 ± 46.18	0.13
Provisc	204.9 ± 26.66	178.9 ± 54.97	0.34
Healon	178.0 ± 15.57	185.3 ± 35.79	0.21
Healon GV	226.3 ± 17.04	184.3 ± 24.50	0.003
HPMC	188.9 ± 29.93	180.6 ± 61.16	0.75
p value	0.002	0.72

**Table 4:** Comparison of stretch ratio in different sub-groups with 90% and 150% energy settings.

Groups	90% Energy	150% Energy	p value
No OVD	2.25 ± 0.15	2.47 ± 0.22	0.03
Viscoat	2.26 ± 0.37	2.42 ± 0.19	0.42
Provisc	2.50 ± 0.09	2.35 ± 0.26	0.20
Healon	2.31 ± 0.23	2.33 ± 0.23	0.99
Healon GV	2.40 ± 0.16	2.29 ± 0.17	0.17
HPMC	2.32 ± 0.15	2.05 ± 0.84	0.63
p value	0.07	0.42	

### SEM imaging

The capsular tags were visible on SEM (Fig. 4G,H). The use of a lower energy setting in the presence of an OVD in the AC, regardless of its type, produced a rougher and a more irregular capsular edge compared to an edge achieved with a higher energy setting (Fig. 4).

## Discussion

In this study we have shown that, increasing the energy from 90 to 150% across the OVD sub-groups, decreased the likelihood of an incomplete laser cut, improved the clinical grading of capsulotomy and circularity, decreased the total arc-length, total tag-length and total tag-area, in addition to reducing the risk of clinically significant tags. Our study results provide an evidence to support the hypothesis of increasing the energy setting, in the presence of an OVD filled AC, to enhance the efficacy of FSL treatment^11^. It suggests that both very high viscosity OVD and differences in refractive index may have an impact on the performance of FSL, but for moderate viscosity OVD, increasing the energy can negate the effects. Amongst the OVDs, Viscoat and Healon GV displayed the worst circularity and other tag-parameters; which were more obvious at the lower energy setting.

Our study showed that both the refractive index and viscosity of an OVD influenced the performance of the FSL. The Z8 FSL used, creates an anterior capsulotomy in a spiral fashion, with a height that is 20 to 60 times higher (400 μm to 600 μm), than the actual thickness of the anterior lens capsule (8 μm to 20 μm)^2,17^. Such a laser profile is used to avoid incomplete laser treatment patterns, (i.e. micro-adhesions/macro-adhesions, non-continuous laser-cuts), and to compensate for any lens-tilt and/or minor intraoperative movements during capsulotomy creation^18,19^. Despite the spiral profile, incomplete capsulotomies occurred with both 90% (40 ± 13.69%) and 150% (20 ± 11.18%) energy settings, in the presence of an OVD. Dick and Schultz also reported the occurrence of incomplete capsulotomies, when they used mechanical pupil expanders with an OVD (Healon) in FLACS^11^.

In order to elucidate these parameters separately, we compared the performance of the laser, in OVDs with the same refractive index, but different viscosity. FSL causes photodisruption and plasma formation of the target tissue. As plasma expands, a cavitation bubble is formed, thus leading to separation of the target tissue^20^. The presence of a viscoelastic fluid has been shown to result in: (1) the formation of fewer and smaller cavitation bubbles; the higher the viscosity, the smaller the size and number^21,22^. (2) A reduction in the expansion and collapse of the cavitation bubble^22^; the viscosity of a fluid exerts a force, which is opposite to the direction of attempted motion in the fluid, thereby dissipating the cavitation bubble’s energy, and slowing down the bubble’s expansion^22^. Hence, increasing viscosity, decreases the maximum bubble radii due to lower bubble energy^22^. (3) A reduction in the speed and force of the collapse, of the laser-induced cavitation bubble, which prolongs the bubble’s lifetime and decreases its impact velocity^22^. A prolonged bubble’s lifetime may also affect the focusing of subsequent laser pulses, reducing their efficiency^23^. Interestingly, even the OVD with the lowest viscosity but same refractive index (i.e. HPMC 2600–7000 mPa)^24^ had a detrimental effect on capsulotomy formation compared to the non-OVD group.

Cohesive OVDs, such as Healon, Healon GV and Provisc, are characterized as being devices with higher viscosity, but with a similar refractive index to aqueous^16,24^. The viscosity of aqueous humour is 1.0 mPa^25,26^, and is 100,000 mPa for Provisc, 300,000 mPa for Healon, and 2,500,000 mPa for Healon GV^24^. Overall, they had worse capsulotomy outcomes than the non-OVD group at 90% energy. Even though Healon performed better at 90% energy compared to Provisc; at 150% energy, the successful creation of a capsulotomy was similar. Hence the higher energy overcame the difference in viscosity between the two OVDs. However, the even higher viscosity of Healon GV resulted in the worse results (circularity, total arc-length, total tag-length and total tag-area) both at 90% and 150% energy settings. Hence the higher energy, even though improved the performance, still resulted in suboptimal outcomes in the presence of Healon GV.

The difference in refractive index (*n*) between aqueous humour and an OVD has been speculated to result in laser beam displacement and affect the cutting-depth range, which may result in an incomplete capsulotomy^10–12,27^. This error in the FSL-capsulotomy depth has been attributed to two plausible factors: (1) the error in ACD measurement by the in-built OCT. (2) The error in FSL beam focus position^16^. Following acquisition of the OCT image, the image is used by the software to calculate the optical path length, which is converted to a physical ACD, by dividing by the refractive index of the aqueous humour (1.336)^16^. A limitation of the software is its inability to compensate for any change in the refractive index. In such a scenario, the ACD reported by the OCT may not be an accurate reflection of the true ACD^16^. Freitas et al. showed that a linear relationship exists between the refractive index of an OVD and the error in OCT-recorded ACD measurement^16^. A change in the refractive index of the AC may also change the optical refraction of the FSL beam, hence the focus position of the beam may be shifted along the axial axis^16^.

The relationship between the refractive index (*n*) of an OVD and the shift in the FSL beam focus position is also linear^16^. Overall, the additive effect of the factors mentioned above can produce an error of − 8 to − 5 μm in the FSL capsulotomy-depth, in the presence of an OVD, with a refractive index (*n*) similar to that of aqueous humour, and up to + 13 μm for Viscoat^16^. However, this calculation was simply based on a mathematical model, by assuming that the contents of the AC were completely replaced by the OVD, and the ACD did not change after the OVD injection, which may not reflect the clinical scenario. The calculation also did not take into account the effect of viscosity. All these factors may underestimate the calculated error. In our study, the presence of Viscoat (refractive index (*n*) = 1.342, in comparison with aqueous humour *n* = 1.336) in the AC, was associated with an irregular laser-cut, along with a distorted capsular edge, when 90% energy was used. A similar trend was observed in the circularity analysis, where Viscoat displayed the worst circularity amongst the 90% energy sub-groups. Using 90% energy, Viscoat displayed a total arc-length and total tag-length that was longer in comparison to other OVDs. The total tag-area achieved with Viscoat was also larger compared to other viscoelastic agents. Nonetheless, the studied capsulotomy parameters improved with an increased energy setting.

The creation of an imperfect FSL-capsulotomy may produce a rough capsular edge and capsular-tags, which are areas of potential weakness and more prone to anterior capsular tears^28^. Daya et al. reported an irregular capsulotomy edge with micro-undulations in a FSL capsulotomy using the Victus system and a cohesive OVD^28^. In our study, capsular tags were evident on SEM across the OVD sub-groups. The use of 90% energy, regardless of the type of an OVD in the AC, created an edge that was rougher and more irregular compared to an edge produced by 150% energy setting. Our SEM findings may be attributed to the OVD affecting the cavitation bubble formation, which was more pronounced at a lower energy setting.

We also compared the effect of the presence of different OVDs on the capsulotomy strength and stretch ratio under two different energy settings. Amongst OVDs, Viscoat had the lowest capsulotomy strength with 90% energy, which could be explained by the presence of irregular laser-cuts, low circularity and worst tag-parameters that were evident in this sub-group. The capsulotomy strength of Viscoat was comparable to the threshold force that has been shown to be required to initiate an anterior capsule tear^28^. The stretch ratios were also comparable amongst the sub-groups with both 90% and 150% energy settings, which could be due to (1) small sample size in each sub-group (2) the sensitivity of the mechanical tester; which might be unable to detect subtle differences in capsulotomy strength and stretch ratio amongst different sub-groups.

Fresh porcine eyes were used in this study, as they are readily available, inexpensive, and have been used in previous studies on femtosecond laser-assisted anterior capsulotomy^7,29–35^. The porcine capsule is more elastic than an adult human capsule^30^. Although similar experiments could be performed in human cadaver eyes, the variation in time from procurement to experimental setup, the effect of storage time on corneal edema and changes in anterior chamber turgidity will induce bias, and considering the large number of globes required for this comparative study, fresh pig eyes were a compromise. We have previously shown that there was no difference in morphological characteristics between capsulotomies created in pig and human eyes^30^. Moreover, the present study focused on the comparisons across the different ophthalmic viscoelastic devices (OVDs) sub-groups. Although the elastic properties of pig capsules are not identical to that of human capsules, the comparisons across the OVDs sub-groups would demonstrate similar trends.

There are a few other considerations in FLACS when the AC is filled with an OVD. The presence of an air bubble in the OVD can disrupt the uniform transmission of the FSL and hence, should be avoided^36^. Although we didn’t experience the collapse of the AC during docking or subsequent laser-treatment, the egress of an OVD can be an issue. This may be avoided by hydrating the paracentesis wound, suture placement or injecting the OVD with a needle into the AC. Lens-tilt due to the insertion of mechanical pupil expanders with an OVD could also affect the precision of FSL-cut^37^. Therefore, it may be worthwhile for the surgeon to adjust the height, safety margins and position of the capsulotomy, which will mitigate the risk of an incomplete laser cut.

The use of a higher energy setting in FLACS may also have negative implications^29,38,39^. Previous studies have suggested that the application of higher energy can denature and melt the collagen fibrils, causing adherence of the capsule and an increased risk of anterior-capsular tags^29^. However, these studies were performed with a higher energy FLACS system (LenSx (Alcon Laboratories, Inc., Forth Woth, TX)^29^. Contrary to most FSL platforms, the LDV Z8 is a low energy (nanojoule range) high-frequency FSL system^2,40,41^. Williams et al. have shown that increasing energy with this laser had a negligible influence on lens-capsule morphology, and strength^31^. Secondly, capsulotomy creation has been implicated as causing an increase in aqueous PGE_2_ levels, seen following FLACS^42^. Recently, Liu et al.^39^ have shown that even when using a low-energy system, a significantly higher aqueous PGE_2_ level was found in comparison to conventional cataract surgery. However, the PGE_2_ increase was significantly lower than that reported with higher energy (μJ) FSL platforms^39^. Future work studying the effects of higher energy on the PGE_2_ level is underway.

In summary, we have determined the effectiveness of FSL-assisted anterior capsulotomy, with the Ziemer LDV Z8, in the presence of different OVDs, in the AC. We have demonstrated that the refractive index and viscosity of an OVD influence the performance of FSL. Overall, increasing energy from 90 to 150% across the OVD sub-groups improved the studied capsulotomy parameters. Therefore, we recommend using a higher energy setting, in the presence of an OVD in the AC, to enhance the efficacy of laser-assisted anterior capsulotomy. We also suggest selecting an OVD with a refractive index similar to aqueous humour and a lower viscosity (e.g. Healon and Provisc). The evidence from this paper shows that with an energy setting of 150% in the presence of an OVD (Healon and Provisc), clinicians can reduce the risk of incomplete laser capsulotomies; capsular tags as well as radial tears.

## Methods

### Experimental groups and Z8 capsulotomy

A total of 96 freshly enucleated porcine eyes (post-mortem time < 8 h), were obtained from a local abattoir (Primary Industries Pte Ltd, Singapore). They were transported, submerged in Optisol (Bausch & Lomb, Inc. USA) to prevent corneal swelling^43^. The corneas were then randomly allocated to one of 12 sub-groups (n = 8).

After corneal epithelial debridement, the porcine eyes were mounted on a holder. A baseline central corneal thickness (CCT) and anterior chamber depth (ACD: defined as the distance between the corneal endothelium and anterior crystalline lens-capsule) were measured with the Visante AS-OCT (Carl Zeiss, Germany)^44^. Three readings were taken, and their average value was recorded (Supplementary Fig. S1A).

A 30-gauge needle was subsequently used to inject 0.30 ml of an OVD in the AC. Five OVDs were used in the present study that included Viscoat (Alcon, Inc. USA), Provisc (Alcon, Inc. USA), Healon (Advanced Medical Optics, Inc. USA), Healon GV (Johnson & Johnson Surgical Vision, Inc. USA) and HPMC (Rayner Intraocular Lenses Limited, UK) with a refractive index (*n)* of 1.342, 1.335, 1.335, 1.337 and 1.335 respectively^16^. The CCT and ACD measurements were repeated to record the change in ACD after the injection of an OVD (Supplementary Fig. S1B).

The porcine eyes were subsequently mounted on a holder, with the suction applied through a 10 ml disposable syringe. A Tonopen (Reichert, Inc. USA) was used to measure the intraocular pressure (IOP) after applying the suction, with the aim to keep the IOP at 12–18 mm Hg. The eyes then underwent anterior capsulotomy with the Ziemer LDV Z8 FSL either in the presence of an OVD in the AC or without. The laser parameters were: 5-mm capsulotomy diameter, 0.8 mm cutting-depth, repetition rate 1 MHz, pulse duration 250 fs, energy 90% or 150%, angulation between the handpiece and Z8 moveable arm − 10°, and suction pressure 400 mbar^40^. After FSL cutting, the CCT and ACD were also measured to see if there was any egress of an OVD during the laser treatment (Supplementary Fig. S1C).

### Evaluation of laser capsulotomy

Following the laser treatment, the porcine cornea was removed, and the continuity of anterior capsulotomy was assessed using a Leica microscope (Leica Microsystems, Germany). A “complete capsulotomy” was defined as a continuous circular opening in the anterior lens capsule. Whereas a laser-capsulotomy was termed as an “incomplete capsulotomy” if there was the presence of (1) micro-adhesions (2) macro-adhesions (capsular tag/tags), or (3) a non-continuous laser-cut.

A utrata forceps (Moria SA, Antony, France Inc.) were subsequently used to remove the anterior lens capsule. The continuity of the laser-cut, the presence or absence of microadhesions/macroadhesions and the ease of extraction of the capsule was used to grade the laser-capsulotomy using a scoring system, from 1 (poor) to 6 (excellent) (Table 5).

**Table 5:** Subjective grading of the quality of capsulotomy.

Grading	Description
1—Poor	Incomplete laser-cut (≥ 10%) with macroadhesions (capsular tag/tags), resulting in very severe resistance to remove the capsule or Irregular laser-cut with significantly distorted capsular edge
2—Bad	Incomplete laser-cut (< 10%) with macroadhesions (capsular tag/tags), resulting in severe resistance to remove the capsule
3—Average	Complete laser-cut and capsule < 50% free with macroadhesions (capsular tag/tags), resulting in moderate resistance to remove the capsule
4—Fair	Complete laser-cut and capsule ≥ 50% but < 90% free with macroadhesions (capsular tag/tags), resulting in mild resistance to remove the capsule
5—Good	Complete laser-cut and capsule ≥ 50% but < 90% free with microadhesion/microadhesions, resulting in minimal resistance to remove the capsule
6—Excellent	Complete laser-cut and capsule ≥ 90% free, with no resistance to remove the capsule or Free-floating capsule

The capsules were then stained with Trypan blue 0.1% (Vision Blue, D.O.R.C. International, Zuidland, the Netherlands) and photographed (× 10 magnification) with a Leica microscope (Leica Microsystems, Germany). The capsular-tags were identified from the photographs of each sample. The explanted lens capsule was then sutured to a membrane of a tissue inserter; with four secure 10-0 Ethicon sutures, for subsequent evaluation by scanning electron microscopy (SEM)^30^. The iris and zonules were cut with scissors to remove the intact nucleus en bloc, and the remaining capsule bag was used for biomechanical testing.

### Evaluation of laser capsulotomy parameters

**Figure 5:**
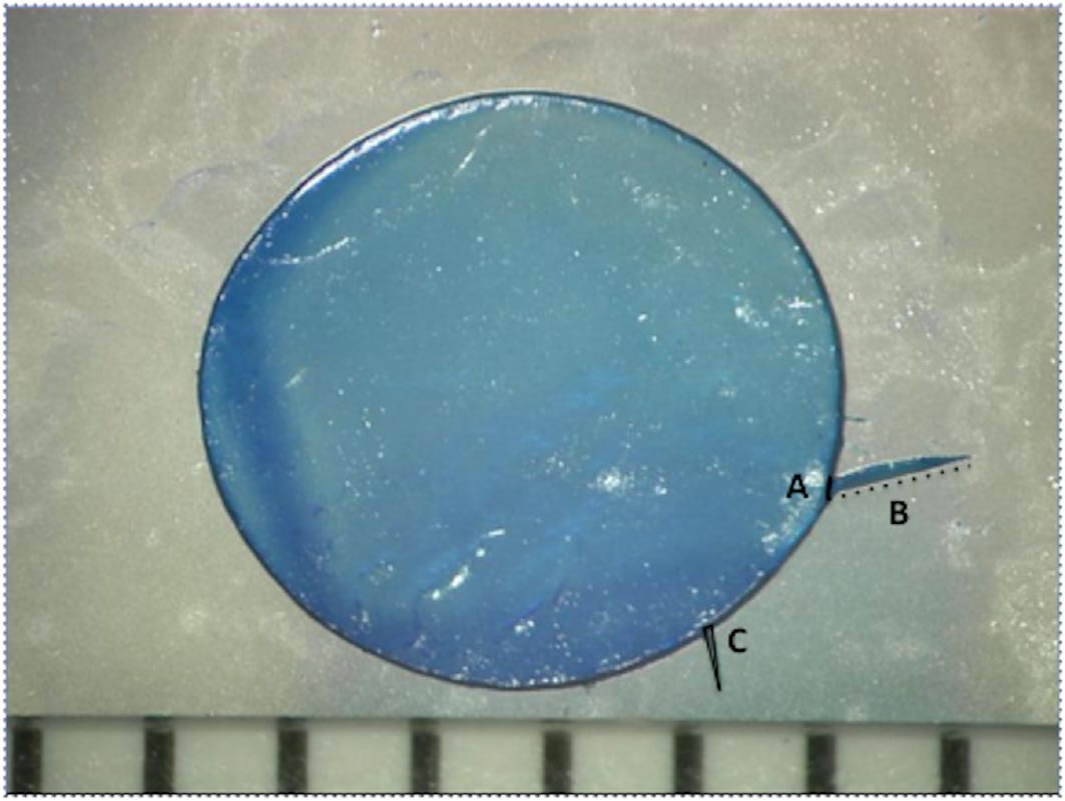
Description of an arc-length, tag-length and tag-area of a capsular tag. (**A**) The arc-length is the distance between the two edges of a capsular-tag, which are attached to the capsule (black line). (**B**) The tag-length is the distance between the edge of a capsular-tag attached to the capsule and the farthest unattached edge (dotted black line). (**C**) The tag-area is the total region of square units occupied by a tag (black triangle).

Image J was used to measure the arc-length of a tag (mm) (defined as the distance between the two edges of a capsular-tag, which are attached to the capsule), tag-length (mm) (defined as the distance between the edge of a capsular-tag attached to the capsule and the farthest unattached edge) and tag-area (mm^2^) (defined as the total region of square units occupied by a tag) by pixilation compared to the diameter of the capsule, which was used as a reference scale (Fig. 5). The total number of capsular-tags in each capsule was recorded.

The measured arc-lengths of the capsular tags were added together to obtain the total arc-length in a capsule. Similarly, the total tag-length and total tag-area were calculated by adding the tag-length and tag-area of each identified capsular-tag, respectively.

Additionally, the circularity of the capsule was determined using the formula 4π × Area/(perimeter)^2^, where a value of 1.0 represented a perfect sphere^45^.

### Scanning electron microscopy (SEM)

SEM was performed to assess morphological imperfections present along the margins of the capsules. All the samples were processed using a previously described technique^46^. Briefly; the extracted porcine capsules were washed twice (for 10 min each) in 1% Phosphate Buffered Saline (PBS). At room temperature, the capsules were immersed in 1% aqueous solution of osmium tetraoxide (FMB, Singapore) for 2 h, and dehydrated in an increasing concentration of ethanol (25%, 50%, 75%, 95% to 100% ethanol). The capsules were air-dried, and carbon adhesive tape was used to mount them on stubs. Subsequently, the capsules were coated with a 10 nm thick layer of gold (Bal-Tec), and their edges were evaluated with a JSM-5600 scanning electron microscope (JEOL, Tokyo, Japan). Four micrographs (1 in each quadrant) were taken under different magnifications (× 140, × 1000 and × 4000).

### Biomechanical testing

The Instron 3343 mechanical tester (Instron Corp, Canton MA) was used to determine the capsulotomy strength of the residual capsular bag using a previously described technique^30^. Briefly; the two mushroom-shaped pins of the apparatus were positioned posterior to the edge of the capsulotomy. The rate of pin displacement was fixed at 6 mm/min. The stretch ratio of the capsulotomy was measured using the formula (capsulotomy size mm + displacement mm)/capsulotomy size mm. In addition, the resistance of the capsulotomy to rupture was calculated in mN.

### Statistical analysis

The statistical analysis was performed with STATA (StataCorp LLC, USA). All data were expressed as mean ± standard deviation. The Kruskal–Wallis test with Dunn’s post hoc test analysed the clinical grading of capsulotomy, circularity, tag parameters and strength parameters in 90% and 150% energy sub-groups. Mann–Whitney U test was used to compare the aforementioned capsulotomy parameters in sub-groups with 90% and 150% energy settings. Pearson correlation analysis was performed to determine the correlation between the studied capsulotomy parameters. The capsular-tags were further dichotomized into “small-tags” and “large-tags” by setting the cut-off value at the mean total arc-length, mean total tag-length and mean total tag-area. A clinically significant tag was defined as a tag with a tag-area of > 0.03 mm^2^.

A Chi-Square test of independence determined whether there was an association between different energy settings (90% and 150%) and the occurrence of an incomplete laser capsulotomy, and the severity of a capsular-tag (arc-length, tag-length and tag-area). A p value of ≤ 0.05 was considered statistically significant.

## Supplementary Information

Supplementary Information.
