# Supplementary material for: Evaluation of femtosecond laser-assisted anterior capsulotomy in the presence of ophthalmic viscoelastic devices (OVDs)
Source: Sci Rep. 2020 Dec 9;10:21542. doi: 10.1038/s41598-020-78361-8 (PMC7726555; doi:10.1038/s41598-020-78361-8)
Supplement: Supplementary file 1 — Supplementary Information. [file 41598_2020_78361_MOESM1_ESM.pdf]

Evaluation of Femtosecond Laser-assisted Anterior Capsulotomy in the Presence of Ophthalmic Viscoelastic Devices (OVDs)

Hassan Mansoor[1,2,4], Yu-Chi Liu [1,2,5], Yoke Rung Wong[3], Nyein C Lwin[1], Xin Y Seah[1],

Jodhbir S Mehta*[1,2,5,6]

[1] Tissue Engineering and Stem Cell Group, Singapore Eye Research Institute, Singapore.

[2] Singapore National Eye Centre, Singapore.

[3] Biomechanics Laboratory, Singapore General Hospital, Singapore.

[4] Al-Shifa Trust Eye Hospital, Rawalpindi, Pakistan.

[5] Ophthalmology Academic Clinical Program, Duke-NUS Graduate Medical School, Singapore.

[6] School of Material Science and Engineering, Nanyang Technological University, Singapore.

Address for correspondence: Prof Jodhbir S Mehta

Singapore National Eye Centre,

11 Third Hospital Avenue, Singapore 168751

E-mail: jodmehta@gmail.com

Supplementary Figure 1:

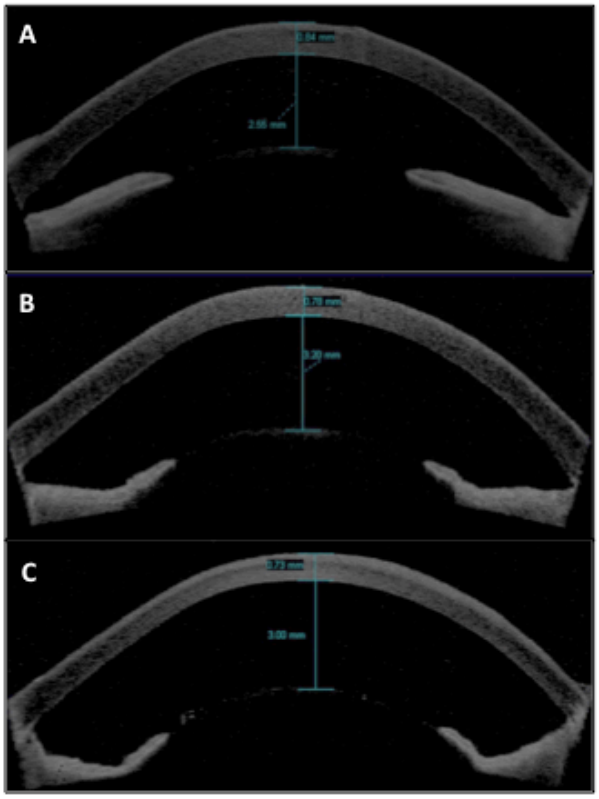

Supplementary Figure 1: Comparison of CCT and ACD during different stages of the experimental setting. (A) Pre-OVD injection (B) Post-OVD injection (C) Post-FSL treatment. The figure shows an increase in the ACD after the injection of 0.30 ml of an OVD. The difference in the ACD between Supplementary Fig 1B and 1C represents the egress of the OVD during the laser treatment.
